# Optimization of Planar Monopole Wideband Antenna for Wireless Communication System

**DOI:** 10.1371/journal.pone.0168013

**Published:** 2016-12-16

**Authors:** Mohammed Nazmus Shakib, Mahmoud Moghavvemi, Wan Nor Liza Mahadi

**Affiliations:** 1 Centre of Research in Applied Electronics, Faculty of Engineering, University of Malaya, Kuala Lumpur, Malaysia; 2 Department of Electrical Engineering, Faculty of Engineering, University of Malaya, Kuala Lumpur, Malaysia; 3 University of Science and Culture, Tehran, Iran; West Virginia University, UNITED STATES

## Abstract

In this paper, a new compact wideband monopole antenna is presented for wireless communication applications. This antenna comprises of a new radiating patch, a new arc-shaped strip, microstrip feed line, and a notched ground plane. The proposed radiating patch is combined with a rectangular and semi-circular patch and is integrated with a partial ground plane to provide a wide impedance bandwidth. The new arc-shaped strip between the radiating patch and microstrip feed line creates an extra surface on the patch, which helps further widen the bandwidth. Inserting one step notch on the ground plane further enhances the bandwidth. The antenna has a compact size of 16×20×1.6mm^3^. The measured result indicated that the antenna achieves a 127% bandwidth at VSWR≤2, ranging from 4.9GHz to 22.1GHz. Stable radiation patterns with acceptable gain are achieved. Also, a measured bandwidth of 107.7% at VSWR≤1.5 (5.1-17GHz) is obtained, which is suitable for UWB outdoor propagation. This antenna is compatible with a good number of wireless standards, including UWB band, Wimax 5.4 GHz band, MVDDS (12.2–12.7GHz), and close range radar and satellite communication in the X-band (8-12GHz), and Ku band (12-18GHz).

## I. Introduction

The recent and anticipated growth of wireless systems has fueled research efforts towards increasing the capacity of wireless systems and its network topology. This increased capacity is the result of both subscriber growth and anticipated data services, which needs significantly higher data rates than voice systems [1–6]. These systems need to be compact and integrated with high performing devices to reduce cost and enhance performance. Thus, it requires wideband antennas for high-speed transmission and simple hardware configuration relative to conventional wireless communication systems [7–10]. UWB (ultrawideband) system is regarded as a promising technology due to its allocation of the 3.1–10.6 GHz bandwidth by the Federal Communications Commission (FCC) [7]. The UWB antenna possesses attractive features, such as low profile, compactness, low-cost, reliability, low-power pulses, and high data transmission. It is also compatible and easily integrated with electronic devices. The current challenge facing engineers is to miniaturize antennas whilst maintaining wideband characteristics. For that purpose, many antenna topologies and configurations for wideband operations have been studied and reported in literature. Different antennas, such as spiral antenna [11] and loop antenna [12] were designed for wideband applications. Several other techniques were introduced, such as Y-V slotted patch [13], and angular folded patch [14]. The designs in [11–14] is capable of achieving wide impedance bandwidth. However, these antennas are relatively larger, which makes it difficult for them to be fitted into small devices.

Planar antennas have received much attention due to its attractive features, such as its compact size, low-profile, light weight, and easy fabrication. Several compact monopole planar antennas with different sizes and shapes are reported in literature. In [15], a linearly tapered slot antenna was compacted by etching one side of the tapered shaped patch and introducing a corrugated pattern of cuts on the right side. This antenna requires an overall size of 36×35×0.8mm^3^. Recently, a monopole antenna is developed using a wrench-shaped feed structure, where the antenna entails a dimension of 20×30×1.6mm^3^ [16]. A printed G-shaped monopole antenna has been proposed for UWB application in [17], where the antenna dimension is 28×35×1.6mm^3^. In [18], a printed antenna with a two-step rectangular radiating patch and a slot inside the patch is introduced with a partial ground plane for the purpose of reducing the ground plane effect and realizing a compactness of 25×26×1.6mm^3^. In [19], a printed monopole antenna with four slots at different corners of a modified radiating patch is developed for UWB application. The antenna occupies a dimension of 25×18×1.6mm^3^. More recently, a monopole antenna with jointed two distinct semi-ellipses through their major axes is developed, where the antenna requires a size of 44×55×0.65mm^3^ in [20]. Another modified semi-circle monopole UWB antenna is achieved with an antenna size of 43×34×1.6mm^3^ [21]. A hexagonal radiating patch with a rectangular slot etched inside the patch and a defected ground plane is designed in [22] for UWB applications. This antenna requires an antenna size of 28×29×1.6mm^3^. In [23], a flag shape monopole antenna fed with an asymmetrically loaded rectangle strip is presented, which requires a compact size of 21.85×28×1.6mm^3^.

In this paper, a new compact wideband antenna is designed. The antenna occupies a small size of 16×20×1.6mm^3^. Utilizing the proposed technique of combining new radiating patch with a new arc-shaped strip, microstrip feed line, and notched ground plane, the antenna achieves a 127% wide impedance bandwidth, ranging from 4.9 to 22.1GHz (at VSWR ≤ 2). The proposed antenna is compact compared to the designs reported in [15–23], which are useful at VSWR ≤ 2. However, they cease to be useful when VSWR is below 1.5, as it does not cover the impedance bandwidth at that range. The measured results showed stable quasi omni-directional radiation patterns. This antenna supports a significant number of wireless communication standards. The proposed antenna achieving 5.1–17 GHz bandwidth (107.7% bandwidth) below the threshold of VSWR 1.5 is suitable for the operations in Direct Sequence at high band (5.8–10.6 GHz) and Multiband OFDM at higher eleven subcarrier bands (5.9–10.6 GHz) for ultrawideband outdoor propagation.

## II. Antenna Design and Structure

**Fig 1:**
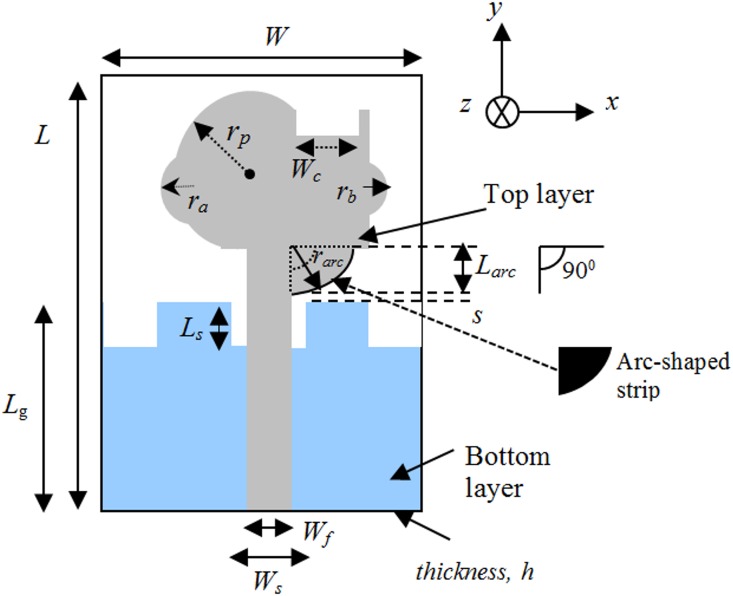
Geometry of proposed patch antenna.

**Fig 2:**
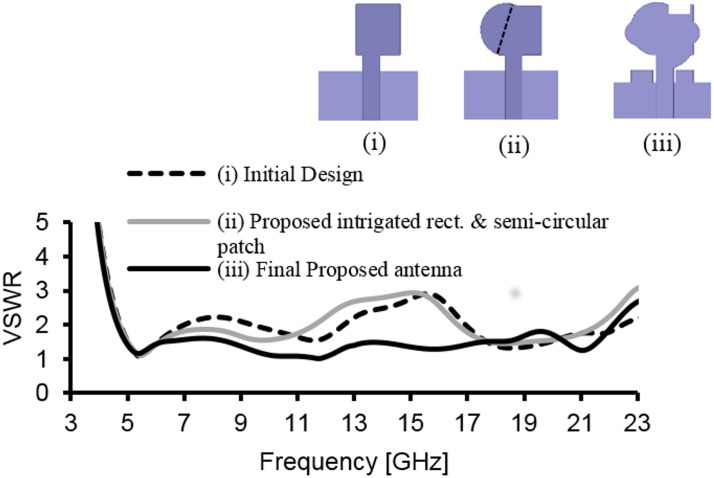
Simulated VSWR of the i) initial design, ii) proposed antenna without arc-shaped strip and ground notch, and iii) Final proposed antenna with arc-shaped strip and ground notch.

**Fig 3:**
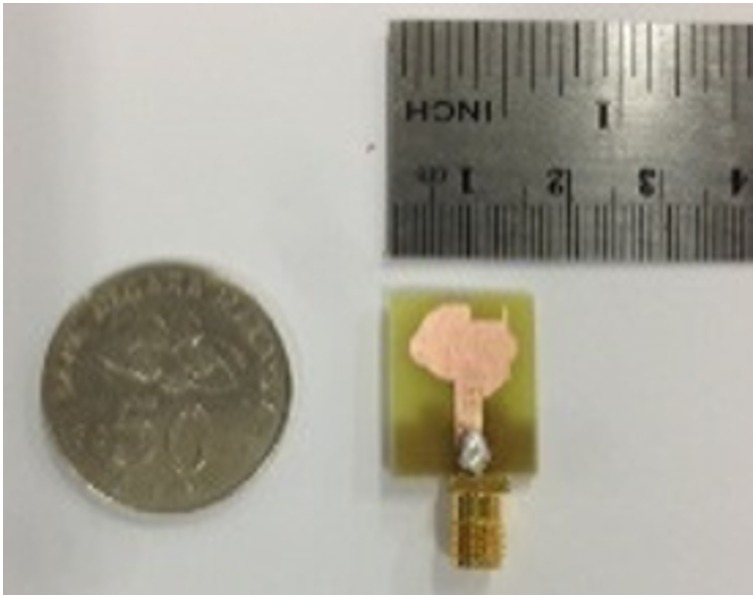
Photo (top view) of the proposed fabricated antenna and shilling for size comparability.

**Fig 4:**
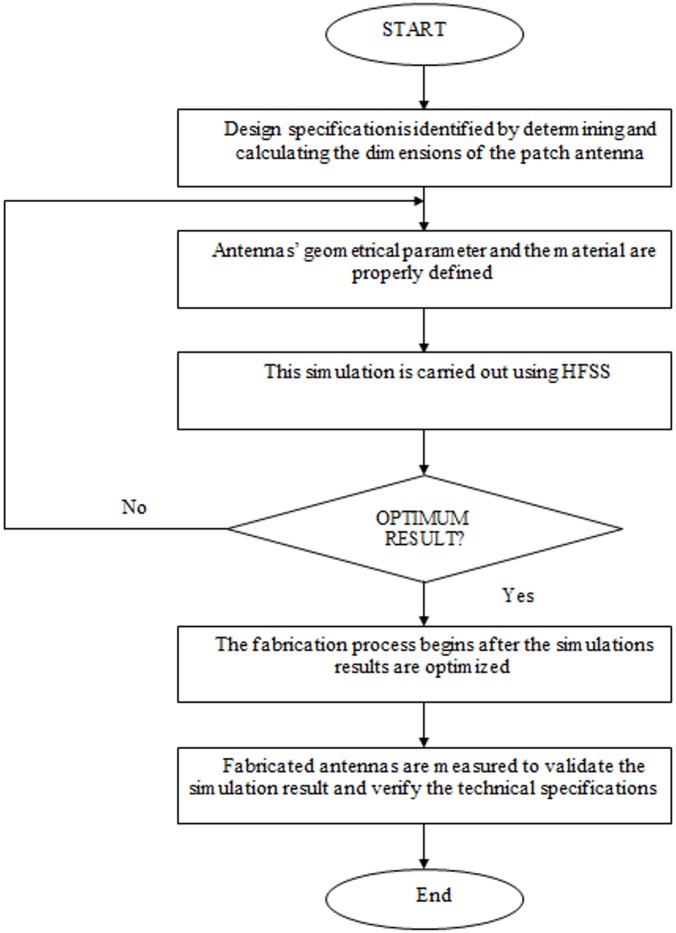
Flow chart of proposed antenna’s design steps.

Fig 1 illustrates the geometry of the proposed antenna. The antenna is fabricated on a FR-4 substrate with a thickness of 1.6 mm, and relative permittivity (*ε*_*r*_) of 4.4. On the top surface, the patch is designed with a combination of a semi-circular and rectangular radiating patch, where the radius of the semi-circular patch is *r*_*p*_. A feed line with a width of *w*_*f*_ is connected to the radiating patch. Fig 2 shows the simulated VSWR of the proposed antenna and proposed antenna without the arc-shaped strip, ground notch and patch slot. As shown in Fig 2, initially (i), the proposed microstrip patch antenna is designed with a rectangular patch and partial ground plane to excite the antenna at three different frequency bands (4.8–6.7GHz, 8.9–12.5GHz and 17–22.2GHz). This ground plane is regarded as an important part of the integrated impedance matching network in the design. Then (ii), a semi-circular patch with radius *r*_*p*_ is combined with the rectangular patch. This helps excite and combine the first two bands to achieve a common wideband of 4.8–10.4GHz, while other band shifts slightly to 16.5-21GHz. Then (iii), a new arc-shaped strip and notched ground plane are introduced to obtain better matching at the 10-16GHz region. This new arc-shaped strip with a radius of 2mm is added between the radiating patch and microstrip line (on the top surface). The arc-shaped is positioned at the right top edge of the feed line, and it acts as an impedance matching element to control the impedance bandwidth of the proposed antenna by creating additional surface current paths in the radiating patch. Hence, the current flow excites and improves the matching on the antenna and holds the frequency below VSWR of 2, especially at the middle frequencies to widen the operating band. This matching is achieved through the optimization in HFSS. The other one-step staircase ground notch is introduced at the feeding position on the ground plane (on the bottom surface,). Therefore, careful adjustment of the parameters of the notch results in better impedance matches. This leads to a wider impedance bandwidth in the proposed design. The two top corner notches on the ground plane produce better matching at higher operating frequency. The microstrip line, including the lower edge portion of the radiating patch and the ground plane close to microstrip line, influence the determination of the lower operating frequencies. A 3.5×1.5mm^2^ rectangular slot 0.5mm away from the top right corner edge of the radiating patch reduces the patch size, as well as improve the bandwidth matching at the higher frequencies. This slot also improves the bandwidth at 6–8.8GHz region to keep the bandwidth close to 1.5 VSWR so that it is suitable for use in close proximity to the human body. The two small circular segments are joined at the left and right portions of the center radiating patch with a radius of *r*_*a*_ and *r*_*b*_, respectively. It helps to obtain better matching at higher operating band. By integrating these techniques, the antenna is excited so that it can obtain a total impedance bandwidth of ~129%, ranging from 4.8–22.2GHz. The optimized parameters of the proposed antenna are: *W* = 16mm, *W*_*c*_ = 3.5mm, *W*_*f*_ = 2.8mm, *W*_*s*_ = 3.7mm, *r*_*p*_ = 4.5mm, *r*_*a*_ = 2mm, *r*_*b*_ = 1.5mm, *r*_*arc*_ = 2.7mm, *L* = 20mm, *L*_g_ = 8mm, *L*_*arc*_ = 2.7mm, *L*_*s*_ = 4mm, *L*_*c*_ = 1.5mm *s* = 0.2mm, and *h* = 1.6mm. A photo of the fabricated antenna is shown in Fig 3. The proposed antenna has a compact dimension of 16 × 20 × 1.6mm^3^. A coin in the figure is used for size comparability. The flow chart of proposed antenna’s design steps is shown in Fig 4.

## III. Result and Discussions

**Fig 5:**
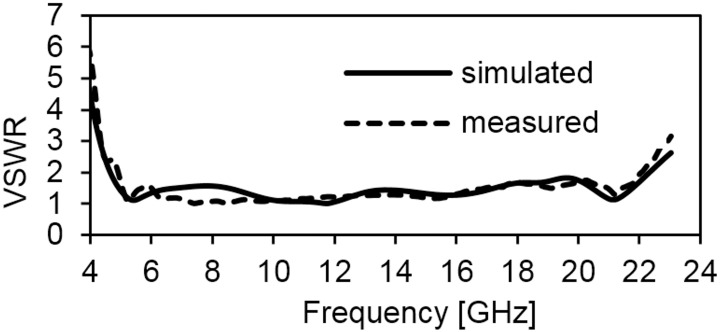
Measured and simulated VSWR of the proposed antenna.

The parameters are optimized using the simulation software HFSS. The fabricated antenna is measured with a Rohde & Schwarz ZVA24 vector network analyzer. Fig 5 shows the simulated and measured results at VSWR ≤ 2; see also S1 Data for more detail. The measured result indicates that the antenna has achieved a wide bandwidth of 17.2 GHz, ranging from 4.9 GHz to 22.1 GHz. The small discrepancy between simulation and measurement results is occurred due to soldering and manufacturing tolerances. The proposed antenna is compact compared to the antennas reported in [6–14]. The proposed antenna is measured and achieved a wide band of 107.7% at VSWR ≤ 1.5, where the operating bandwidth covers 5.1–17 GHz. It is used to operate the antenna below the VSWR of 1.5 threshold levels, as the acceptable operating bandwidth can be used significantly while placing the antenna close to a human tissue/body, metal device, or any metal desk. Moreover, this type of specifications is allocated for outdoor propagation in the UWB system. Therefore, the proposed antenna impedance bandwidth of 5.1–17GHz is suitable for operation in the Multiband OFDM and Direct Sequence high bands for UWB outdoor propagation. The proposed monopole antenna is optimized by maximizing the Effective Bandwidth of Interest per unit Volume (EBIV) of the antenna, as indicated in [24].

$$\text{EBIV }=\;\frac{\text{Total Bandwidth }\left(\text{GHz}\right)}{\text{Volume of entire structure }\left({\text{cm}}^{3}\right)}\;\times \%\text{BW }\times \text{ Mean efficiency}\%$$ (1)

In this paper, the comparison is made with adopting the relation of EBIV index and antenna volume with several wideband antennas. The volume occupied by the proposed monopole antenna is 0.512cm^3^ when using the antenna dimension stipulated in Fig 1. By adding the simulated efficiency and the measured impedance bandwidth of 0.77 and 1.27, respectively, the EBIV index is ~32.85. Thus, the proposed monopole antenna has a higher figure of merit of EBIV index compared to the reported monopole antennas in [15–23]. The comparison of the EBIV of different recently reported monopole antennas in [15–23] are given in Table 1.

**Table 1:** Comparison of Different Antennas EBIV Index.

Ref.	Volume (cm^3^)	VSWR≤2 TotalBandwidth (GHz)	BW (%)	Bandwidth Range (GHz)	Radiation Efficiency (%)	EBIV(GHz/cm^3^)
[15]	1.008	7.5	109.5	3.1–10.6	100*	8.15
[16]	0.960	10.9	127.5	3.1–14	80	14.48
[17]	1.568	8.0	110	3–11	100*	5.612
[18]	1.034	10.3	123.4	3.2–13.5	100	12.29
[19]	0.72	9.75	122	3.1–12.85	100*	16.52
[20]	1.573	8.9	135.9	2.1–11	100*	7.69
[21]	2.339	8.4	131.3	2.2–10.6	81	2.34
[22]	1.299	5.07	61.34	5.73–10.8	100*	2.39
[23]	0.979	8.9	117.9	3.1–12	85	14.48
Proposed	0.512	17.2	127	4.9–22.1	77	32.85

*efficiency not given. Ideal case, efficiency is 100%

**Fig 6:**
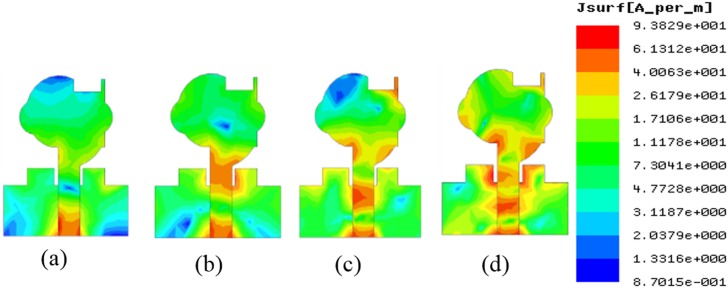
Simulated current distribution at a) 7GHz, b) 10GHz, c) 15GHz, d) 21GHz.

**Fig 7:**
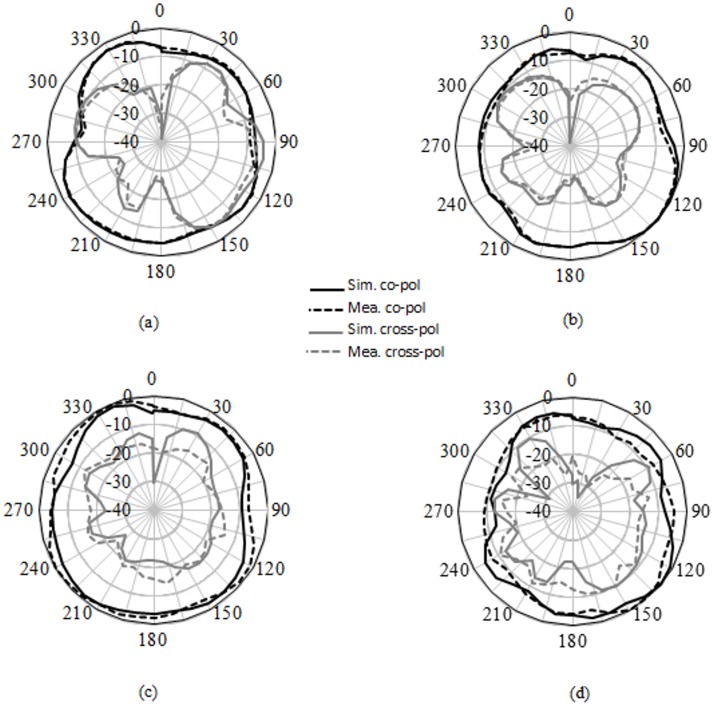
Simulated and measured radiation pattern. a) *xz*-plane at 4.9GHz, b) *yz*-plane at 4.9GHz, c) *xz*-plane at 7GHz, d) *yz*-plane at 7GHz.

**Fig 8:**
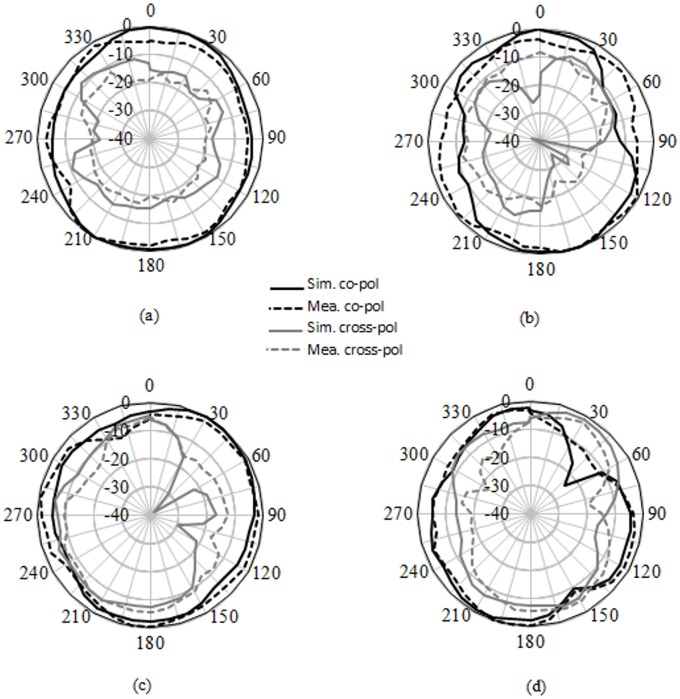
Simulated and measured radiation pattern. a) xz-plane at 10GHz, b) yz-plane at 10GHz, c) xz-plane at 15GHz, d) yz-plane at 15GHz.

**Fig 9:**
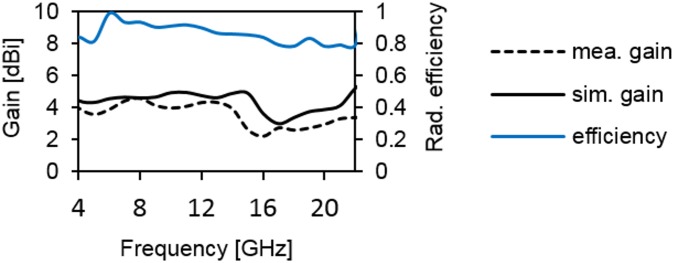
Gain and efficiency of the proposed antenna.

**Fig 10:**
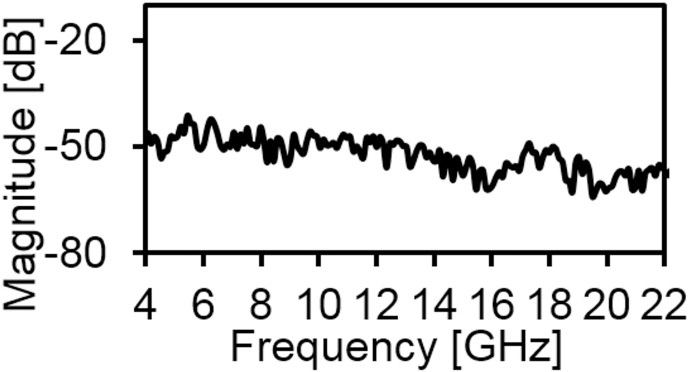
Measured S_21_ (Magnitude) of the proposed antenna.

**Fig 11:**
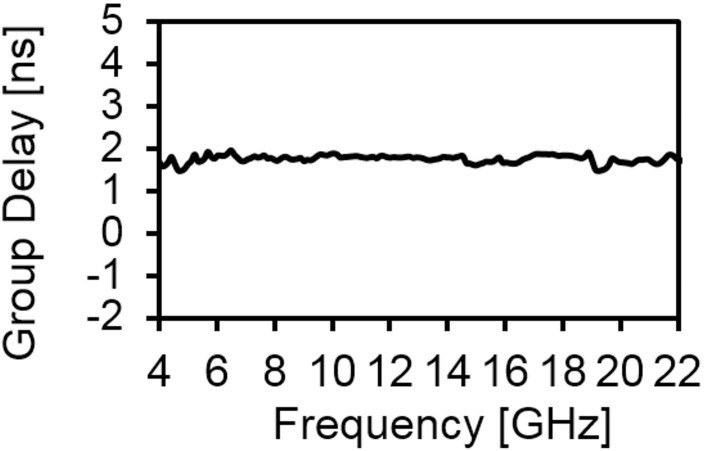
Measured group delay of the proposed antenna.

**Fig 12:**
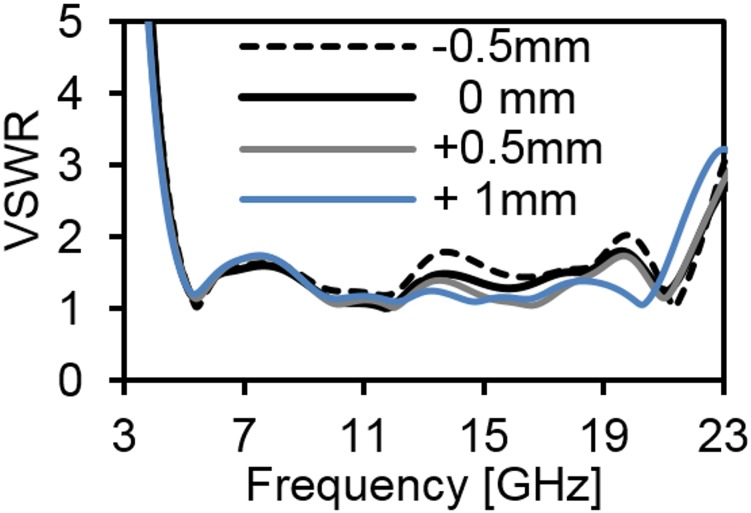
Effects on VSWR with changing *s* parameter.

**Fig 13:**
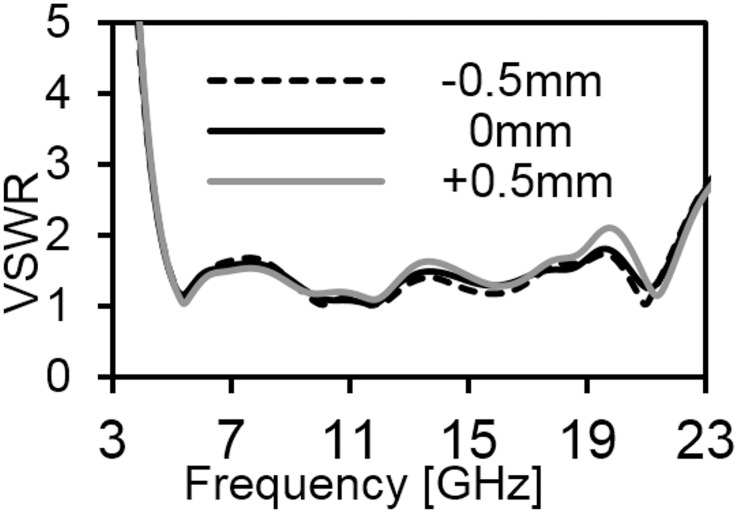
Effects on VSWR with changing *r*_*arc*_ parameter.

**Fig 14:**
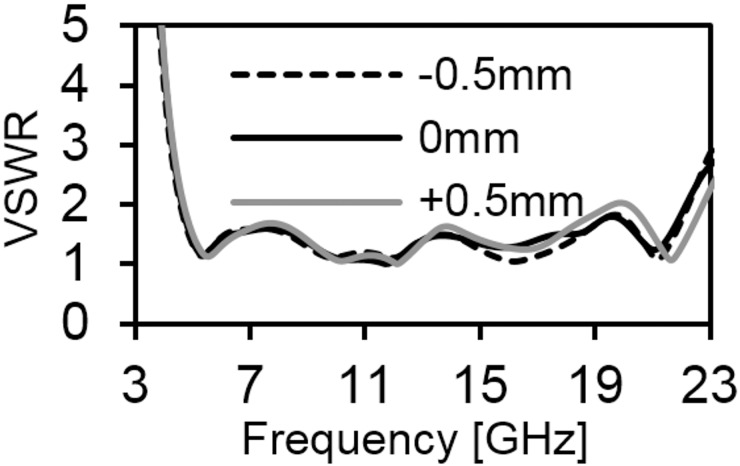
Effects on VSWR with changing *r*_*p*_ parameter.

Fig 6 provides the current distribution of the antenna. At 7 GHz, a concentrated current flows on the lower edge of the radiating patch. At 10 GHz, a strong current excites the notched ground plane, feed line, and arc-shaped strip. This suggest that the arc-shaped strip, notch ground plane, and lower portion of the radiating patch plays a significant role in achieving a wide impedance bandwidth. It can also be observed that the electric currents are concentrated around the feeding strip. Thus, the ground plane significantly affects the impedance radiation performance of the antenna. At 15 GHz, the concentrated current flows from the feed line to the lower portion of radiating patch, radiating patch strip, and arc- shaped strip. Again, at 21GHz, a strong current is distributed on the notch ground plane, feed line to the radiating patch, corner of arc-shaped strip, *r*_*a*_, *r*_*b*_ as well as on the radiating patch slot. It is observed that a significant amount of current is distributed on the patch at all frequencies. In addition, the concentrated current is observed on the antenna’s ground plane at all operating frequencies, which confirms the significant effects on the antenna performance. The measured radiation patterns at 4.9 GHz and 7 GHz are shown in Fig 7. As indicated in the figure, the antenna shows omnidirectional radiation pattern in both *xz*- and *yz*-planes. At 10 GHz and 15 GHz (in Fig 8), the antenna observes omnidirectional and quasi-omnidirectional patterns at *xz*-plane and *yz*-plane, respectively. At higher band, antenna at both planes observes quasi-omnidirectional radiation patterns. The peak gain and radiation efficiency of the antenna is shown in Fig 9. As shown in the figure, the antenna operates a gain above 3.58 dBi at the UWB band and drops at the higher band. The radiating efficiency is above 77% over the entire operating band. To investigate the transfer function consistency in the frequency-domain, the antennas are examined in a face-to-face scenario. In this scenario, two identical antennas are placed at a distance of 50 cm. The measured magnitude of S_21_ is shown in Fig 10. In Fig 11, the measured group delay is observed. As shown in the figure, the group delay variation is less than 0.49 ns over the entire operating band. A parameter study has been performed to observe the impedance matching of the antenna. In the simulation of the antenna, when one parameter changes, the rest of the parameters are kept similar to the optimization parameters listed in Table 1. Fig 12 shows the variation of *s* on the proposed antenna. By increasing *s* to +1mm, a matching at lower frequencies is occurred. Although, a better matching occurred at higher frequencies, a bandwidth reduction results at the higher end band. Again, with decreasing *s*, a mismatch occurs on the middle resonant frequencies and shifts upwards, resulting in a bandwidth reduction on the antenna. Hence, *s* = 0.2mm was chosen as the control model. Fig 13 shows the variation of *r*_*arc*_ on the proposed antenna. With decreasing *r*_*arc*_, the operating bandwidth remains the same. However, with increasing *r*_*arc*_, a mismatch occurs on the upper resonant frequency. The higher frequency band shifts upward, and as a result of this, bandwidth reduction is observed. Hence, *r*_*arc*_ = 2.7mm was chosen as the control model in the design. Fig 14 shows the variation of *r*_*p*_ on the antenna. With decreasing *r*_*p*,_ the bandwidth remains same. But with increasing *r*_*p*_, the operating band decreases as the frequencies at the upper band shifts upwards. Hence, *r*_*p*_ = 4.5mm was chosen as the control model.

## IV. Conclusion

In this paper, a new printed compact antenna for wideband application has been proposed and investigated. Employing a new radiating patch technique with notched ground plane and a new arc-shaped strip resulted in the development of a broadband antenna with low profile characteristics. The antenna is optimized to achieve wideband performance. The measured results indicated that the antenna has obtained an impedance bandwidth of 127% (4.9–22.1 GHz) at VSWR ≤ 2 and 107.7% (5.1–17 GHz) at VSWR ≤ 1.5. The designed antenna has a simple configuration, with a compact size of 16×20×1.6 mm^3^. Stable gain with omni-directional radiation patterns has been observed. The proposed antenna is a suitable candidate for wireless communication systems, especially for indoor/ outdoor UWB applications and also for Wimax 5.4 GHz band, MVDDS (12.2–12.7 GHz), and close range radar and satellite communication in the X-band (8–12 GHz), and Ku band (12–18 GHz).

## Supporting Information

S1 Data VSWR simulated and measured data. (XLSX)
